# Haemocytes collected from experimentally infected Pacific oysters, *Crassostrea gigas*: Detection of ostreid herpesvirus 1 DNA, RNA, and proteins in relation with inhibition of apoptosis

**DOI:** 10.1371/journal.pone.0177448

**Published:** 2017-05-18

**Authors:** Claire Martenot, Ophélie Gervais, Bruno Chollet, Maryline Houssin, Tristan Renault

**Affiliations:** 1 Ifremer (I*nstitut Français de Recherche pour l'Exploitation de la Mer)*, *Laboratoire de Génétique et Pathologie des Mollusques Marins*, *La Tremblade*, *France*; 2 LABEO Frank Duncombe, Caen, France; 3 Ifremer, *Département Ressources Biologiques et Environnement*, *Nantes*, *France*; Bigelow Laboratory for Ocean Sciences, UNITED STATES

## Abstract

Recent transcriptomic approaches focused on anti-viral immunity in molluscs lead to the assumption that the innate immune system, such as apoptosis, plays a crucial role against ostreid herpesvirus type 1 (OsHV-1), infecting Pacific cupped oyster, *Crassostrea gigas*. Apoptosis constitutes a major mechanism of anti-viral response by limiting viral spread and eliminating infected cells. In this way, an OsHV-1 challenge was performed and oysters were monitored at three times post injection to investigate viral infection and host response: 2h (early after viral injection in the adductor muscle), 24h (intermediate time), and 48h (just before first oyster mortality record). Virus infection, associated with high cumulative mortality rates (74% and 100%), was demonstrated in haemocytes by combining several detection techniques such as real-time PCR, real-time RT PCR, immunofluorescence assay, and transmission electron microscopy examination. High viral DNA amounts ranged from 5.46×10^4^ to 3.68×10^5^ DNA copies ng^-1^ of total DNA, were detected in dead oysters and an increase of viral transcripts was observed from 2, 24, and 48hpi for the five targeted OsHV-1 genes encoding three putative membrane proteins (ORFs 25, 41, and 72), a putative dUTPase (ORF 75), and a putative apoptosis inhibitor (ORF 87). Apoptosis was studied at molecular and cellular levels with an early marker (phosphatidyl-serine externalisation measured by flow cytometry and epifluorescence microscopy) and a later parameter (DNA fragmentation by terminal deoxynucleotidyltransferase-mediated dUTP nick end labeling assay (TUNEL)). The down-regulation of genes encoding proteins involved in the activation of the apoptotic pathway (TNF and caspase 3) and the up-regulation of genes encoding anti-apoptotic proteins (IAP-2, and Bcl-2) suggested an important anti-apoptosis phenomenon in haemocytes from OsHV-1 infected oysters at 24 and 48hpi. Additionally, more phosphatidyl-serines were externalized and more cells with DNA fragmentation were observed in haemocytes collected from artificial seawater injected oysters than in haemocytes collected from OsHV-1 infected oysters at 24 and 48hpi, suggesting an inhibition of the apoptotic process in presence of the virus. In conclusion, this study is the first to focus on *C*. *gigas* haemocytes, cells involved in the host immune defense, during an OsHV-1 challenge in controlled conditions by combining various and original approaches to investigate apoptosis at molecular and cellular levels.

## Introduction

During the last decade, mass mortality outbreaks of young Pacific oysters, *Crassostrea gigas*, have been regularly reported each year since 2008 in France and throughout the world, in association with the detection of particular genotypic variants of ostreid herpesvirus type 1 (OsHV-1). This virus is one of the major pathogens affecting *C*. *gigas* and constitutes the type species within the genus *Ostreavirus* and the *Malacoherpesviridae* family [1–3]. OsHV-1 variants had been reported and a variant named μVar was mainly detected along the French coast [4–9]. Molecular investigation allowed the description of OsHV-1 variants in Ireland [10–12], Spain [13–15], Italy [16,17], Portugal [18], South Korea [19,20], United States [21–23], Mexico [24], Australia [25–28], and New Zealand [8,29–31] during mortality outbreaks or without oyster mortality. To better understand this large distribution of OsHV-1 around the world and its replication cycle, it is important to bring data related to the interactions between the virus and its host, particularly at the cellular level. Contrary to vertebrates, there is no immunological memory after an initial response to a specific pathogen in relation to the production of antibodies and specific T cell response. Even if anti-viral immunity in molluscs remains poorly characterized, recent transcriptomic approaches revealed that several defense-related oyster transcripts were induced in OsHV-1 infected spat and lead to the assumption that the innate immune system plays a crucial role against the virus [32–36]. Among the innate immune defense in invertebrates, programmed cell death (i.e. apoptosis and autophagy) constitutes a major mechanism of anti-viral response by limiting viral spread and eliminating infected cells, especially in marine molluscs [33,37–42]. Apoptosis can be initiated in cells through either the intrinsic (mitochondrial-mediated) and extrinsic (stimulation of transmembrane death receptors) pathways. The activation of the extrinsic apoptosis pathway by the interaction of the tumor necrosis factor (TNF) with its receptor (TNFR), results in the Fas-associated death domain protein (FADD) and caspase-8 (casp-8) recruitment and then activates the caspase-3 (casp-3), which plays a central role in the apoptosis execution phase, conducting to the fragmentation of the DNA [43]. Components of the extrinsic apoptosis pathway were induced in response to OsHV-1 infection in oyster gills [39]. In addition, autophagy was induced in the mantle of oysters in response to OsHV-1 infection and conferred a protective role against the virus [41]. A homolog to the anti-apoptotic protein, B*-*cell lymphoma 2 (Bcl-2), involved in the intrinsic apoptosis pathway, was reported from Pacific oyster haemocytes exposed to OsHV-1 during *in vitro* assays [33] and in the Mediterranean mussels, *Mytilus galloprovincialis* [44].

Some viruses, including herpesviruses, have developed different strategies to evade or module host apoptosis and facilitate viral replication, spread, and persistence/latency [37,45–50]. OsHV-1 may actively manipulate host apoptosis since several viral genes (ORFs 42, 87, 99, and 106) encoding putative apotposis inhibitors (IAP) were highly expressed in mantle and gills during the acute stage of infection, facilitating virus infection [35,36,39].

The objective of the present study is to better understand the apoptosis response in *C*. *gigas* oysters during an OsHV-1 experimental infection at molecular (gene expression) and cellular (cytoplasmic membrane modification and DNA fragmentation) levels. In this way, the RNA expression of five host genes corresponding to proteins involved in the apoptotic pathway (Bcl-2, IAP-2, TNF-2, TNFR, and casp-3), were monitored at three times post infection: 2h (early after viral injection in the adductor muscle), 24h (intermediate time), and 48h (just before first oyster mortality record). Apoptosis was studied at cellular level with an early marker (phosphatidyl-serine externalisation measured by flow cytometry and epifluorescence microscopy) and a later parameter (DNA fragmentation by terminal deoxynucleotidyltransferase-mediated dUTP nick end labeling assay (TUNEL)), as previously used by Gervais *et al*. [51] to investigate apoptosis in flat oyster, *Ostrea edulis*. Contrary to the main previous studies, the originality of the present investigation was focused on haemocytes, from experimentally infected and control oysters, which play a key role in the oyster immunity [52,53]. In addition, virus infection associated with high mortality rates was demonstrated by OsHV-1 DNA, RNA, protein, and viral particle detection in haemocytes.

## Results

### Mortality monitoring and DNA quantification in oyster mantle and haemocytes

**Fig 1:**
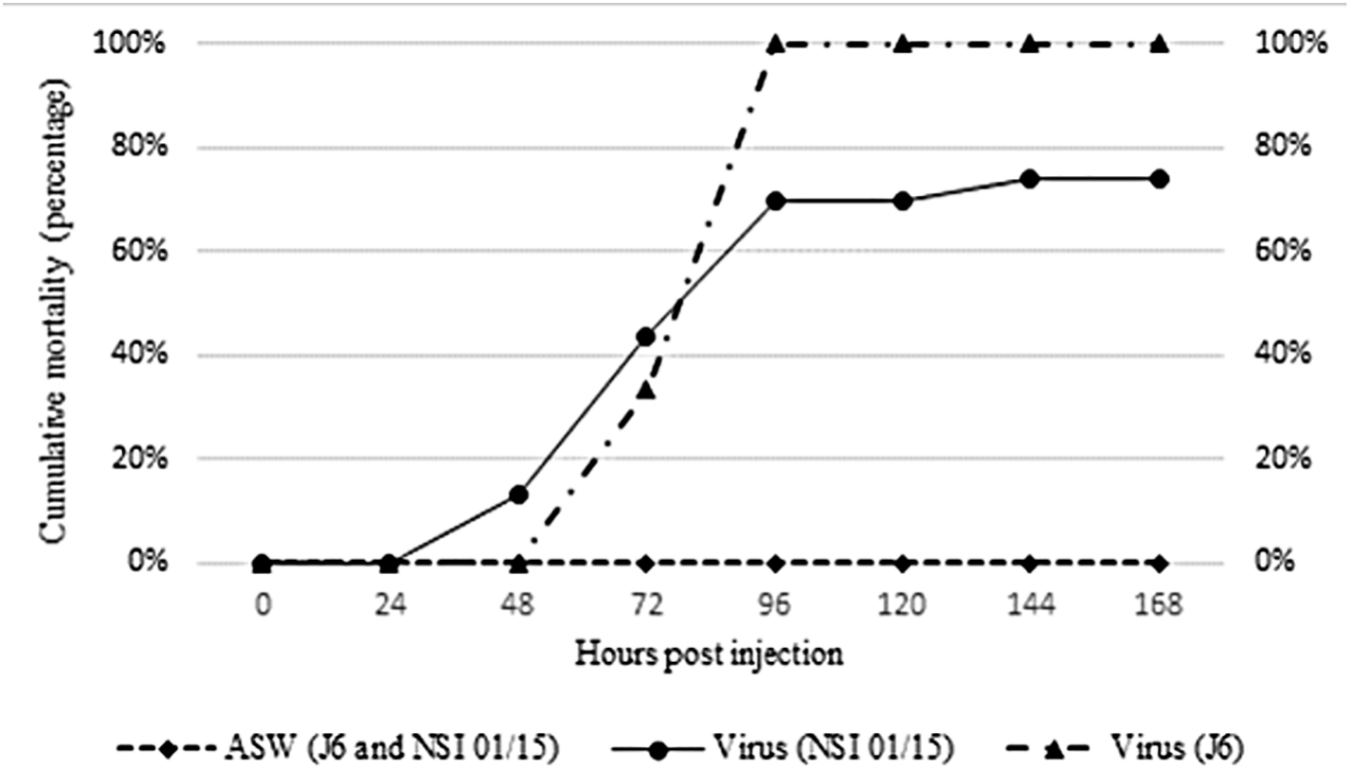
Cumulative mortality of Pacific oysters (NSI 01/15 and J6) experimentally infected by OsHV-1 or injected with sterilized artificial sea water (ASW) during one week (168h).

Oyster mortality was first observed at 48hpi and increased gradually from 48hpi to 96hpi for the two batches, regardless of the animal age (Fig 1). However, the final mortality rate was significantly different between the NSI 01/15 (74%; 17/23) and J6 (100%; 9/9) (Fig 1). No mortality was recorded in control tanks containing oysters injected with ASW.

**Fig 2:**
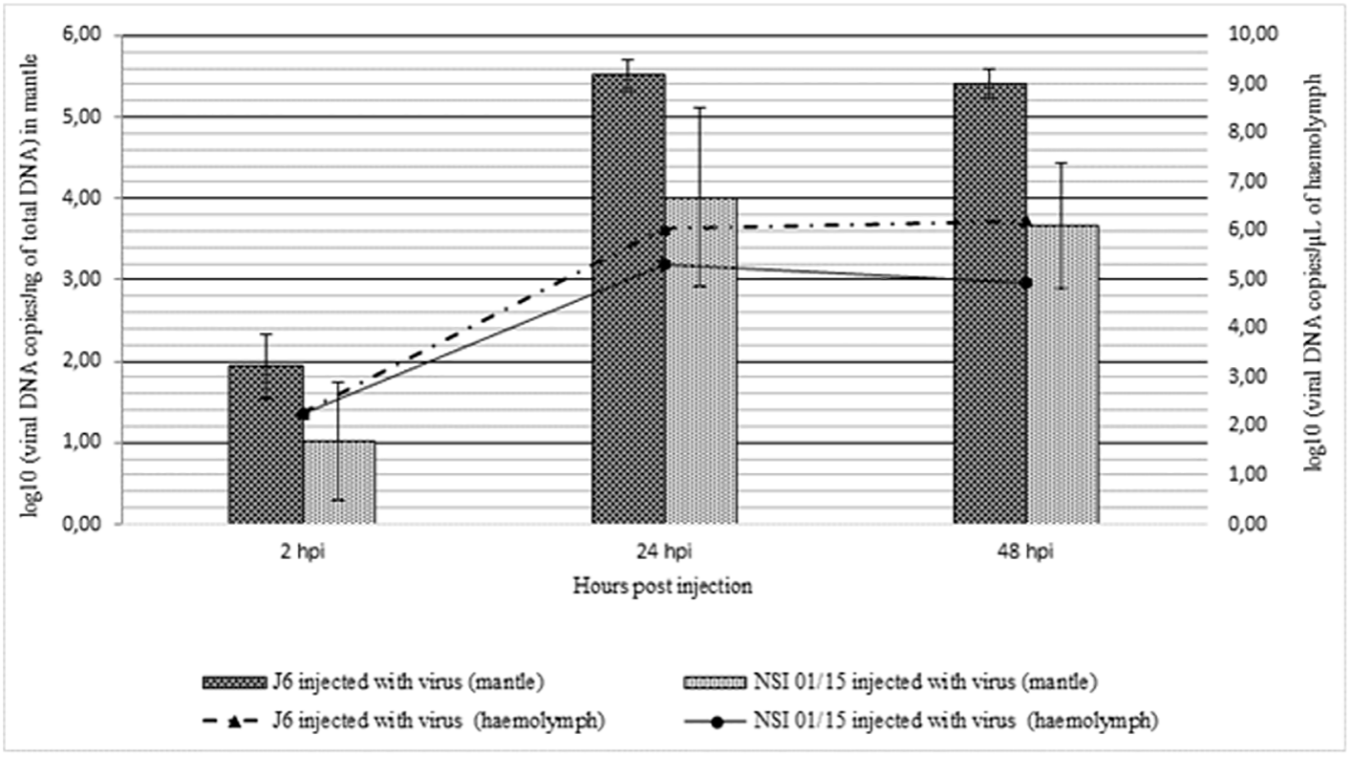
Viral DNA quantification in haemolymph and in the mantle of oysters (five animals per sampling time) infected by OsHV-1 at 2, 24, and 48 hours post injection (hpi).

No significant difference was observed between the two infected oyster batches at 2hpi (p = 0.128), 24hpi (p = 0.100), and 48hpi (p = 0.100) for the OsHV-1 DNA amounts (Fig 2). No viral DNA was present in the mantle of NSI 01/15 oysters injected with ASW whereas OsHV-1 DNA was detected below the quantification limit of the real time PCR in the mantle and the haemolymph of J6 oysters injected with artificial seawater (data not shown).

### OsHV-1 transcript detection in haemocytes at different times post viral injection

**Fig 3:**
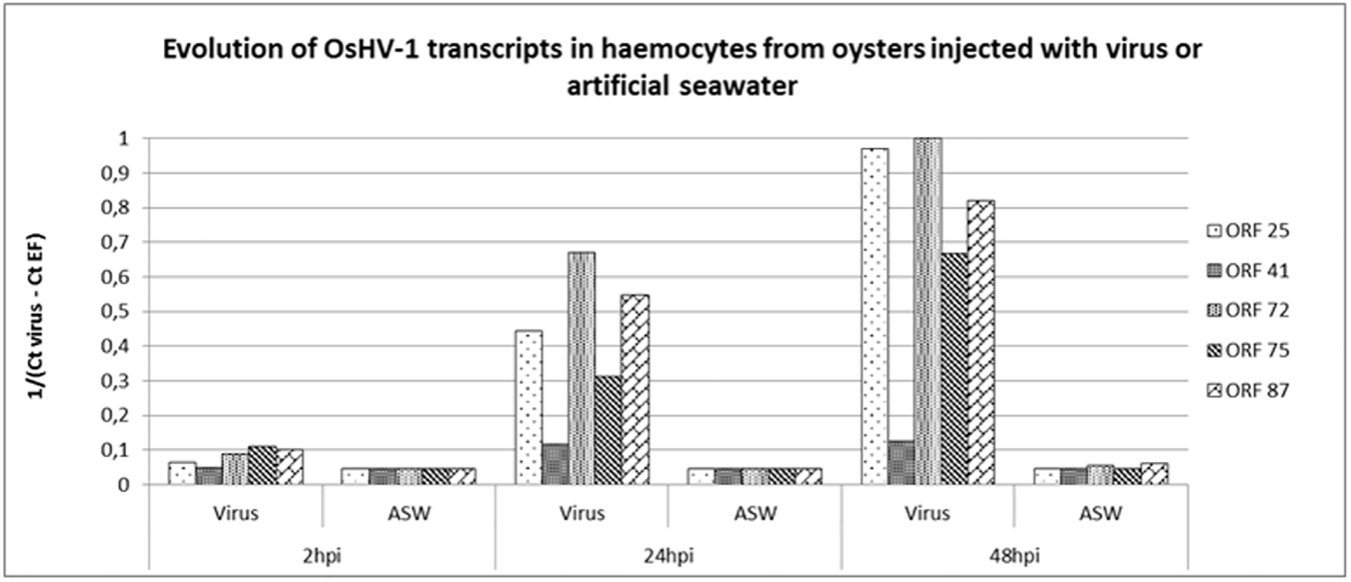
OsHV-1 transcript detection in haemocytes from oysters injected with virus or artificial seawater (ASW) at 2, 24, and 48 hours post injection (hpi).

For all targeted viral ORFs (25, 41, 72, 75, and 87), the OsHV-1 transcript number was higher in haemocytes from infected oysters than from oysters injected with ASW, especially at 24 and 48hpi (Fig 3). In addition, the viral transcript number strongly increased from 2hpi, to 24hpi, and 48hpi in haemocytes from experimentally infected oysters except for the ORF41 (Fig 3).

### Viral protein and capsid detection in haemocytes

**Fig 4:**
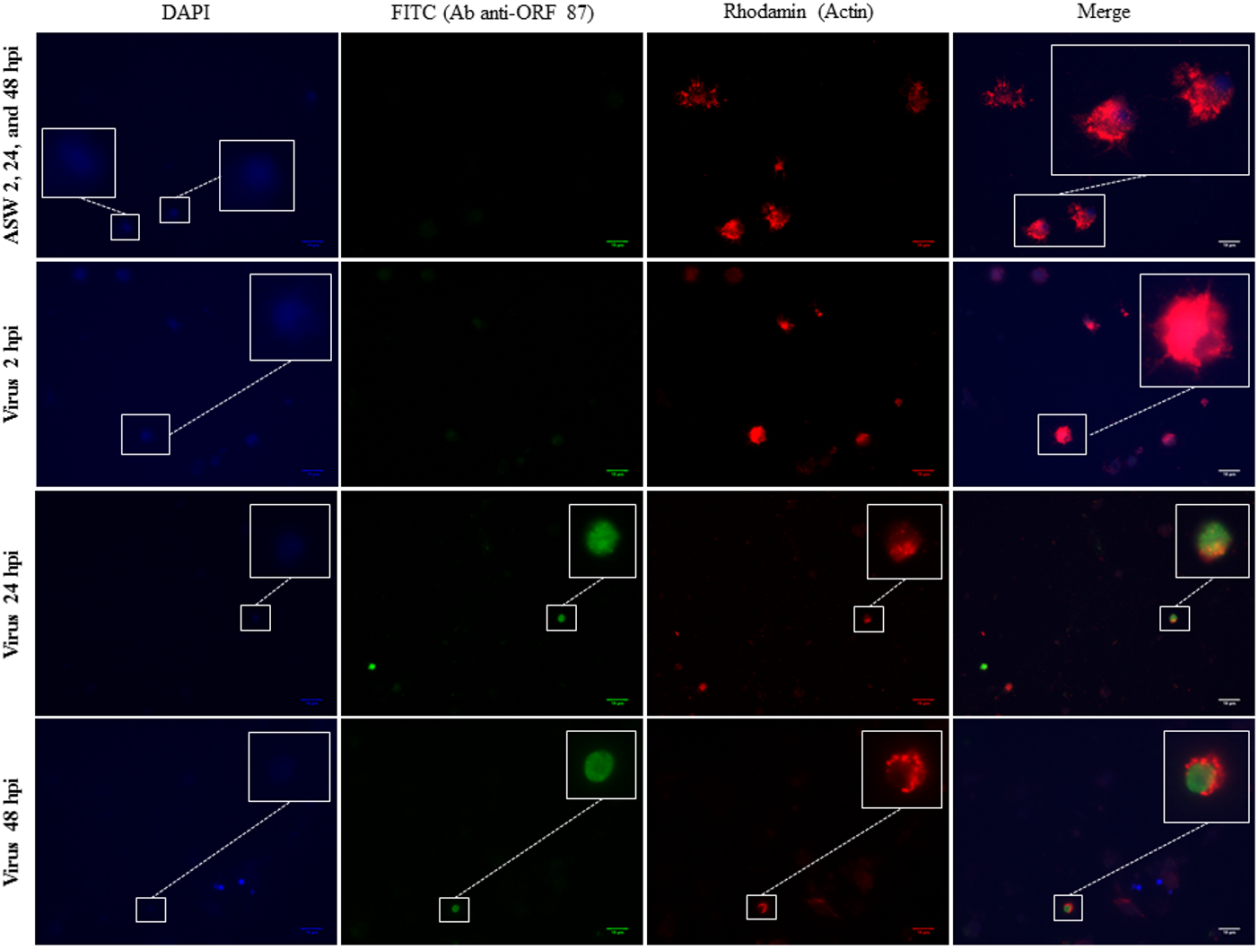
Double immunofluorescence assay using antibodies against a viral putative apoptosis inhibitor (FITC) and actin (rhodamin) in haemocytes from OsHV-1 infected and control oysters at 2, 24, and 48hpi. Bar = 10μM.

A double fluorescence labelling was performed for the detection of viral proteins and actin in haemocytes from OsHV-1 infected and ASW injected oysters at 2, 24, and 48hpi. Viral proteins were targeted using the polyclonal antibody against a putative apoptosis inhibitor encoded by ORF 87 (Fig 4). No viral protein was detected in haemocytes from oysters injected with ASW and OsHV-1 infected oysters sampled at 2hpi (Fig 4). Viral proteins were detected in haemocytes from OsHV-1 infected oysters collected at 24 and 48hpi (Fig 4).

**Fig 5:**
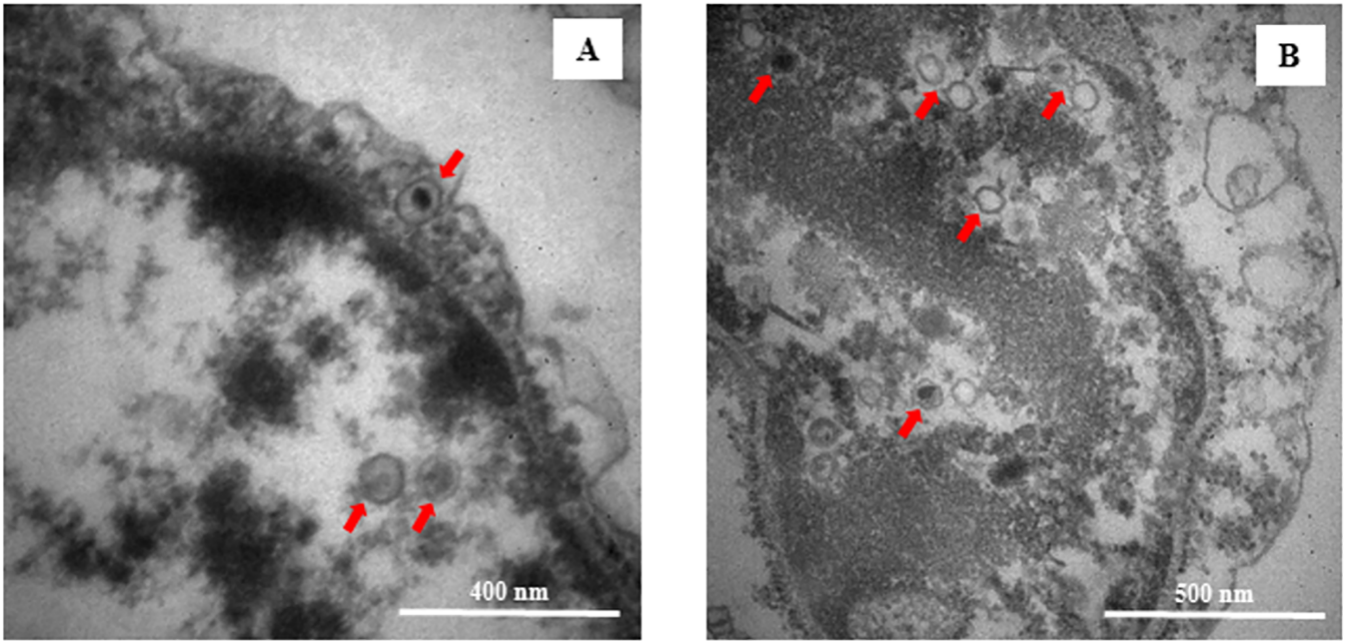
Transmission electron micrograph of haemocytes or fibroblast-like cells from OsHV-1 infected oysters at 24h (A) and 48h (B) post viral injection in the adductor muscle. Arrows indicate viral nucleo-capsids or empty viral capsids.

To complete OsHV-1 DNA, RNA, and protein detection in haemocytes, TEM examination was especially performed at 24 and 48hpi in haemocytes from infected oysters (Fig 5). Viral capsids were observed in the nucleus of very few cells interpreted as haemocytes (Fig 5).

### Apoptosis modulation at cellular level: Phosphatidyl-serine externalization and DNA fragmentation

**Fig 6:**
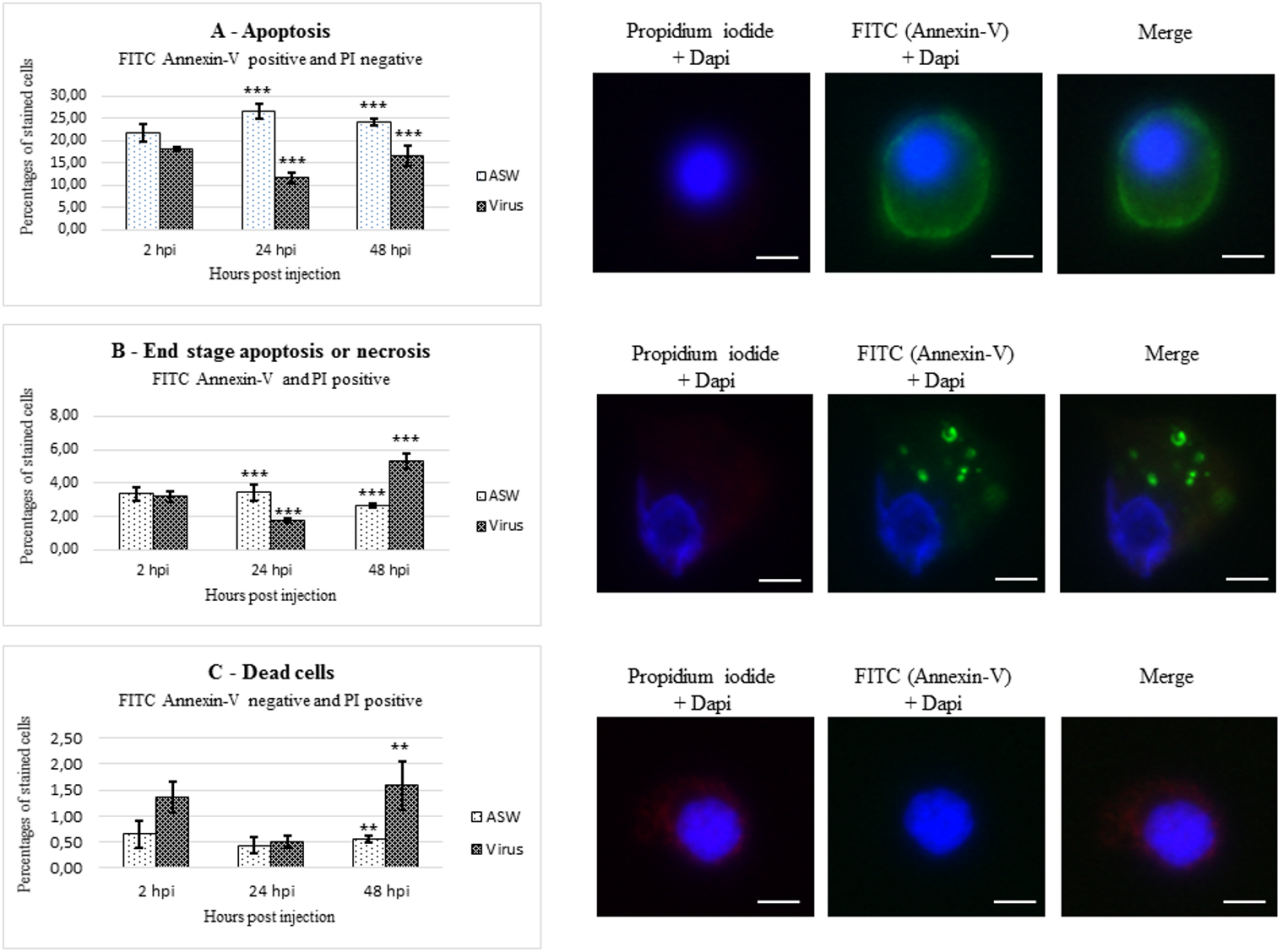
Percentages of early apoptotic (A), late apoptotic/secondary necrotic (B), and primary necrotic/dead cells (C) in haemocytes from OsHV-1 infected (virus) and control oysters (ASW) at 2, 24, and 48hpi. **P<0.01, ***P<0.001. Bar = 5μM. Each apoptosis stage was illustrated by fluorescence microscopy figures (dapi = cell nucleus).

Percentages of apoptotic, necrotic, and dead cells in haemocytes from OsHV-1 infected and ASW injected oysters were represented in the Fig 6.

At 2hpi, no significant difference was reported between haemocytes from OsHV-1 infected and ASW injected oysters for the percentages of early apoptotic (p = 0.133), late apoptosis/secondary necrotic (p = 0.992), and primary necrotic/dead cells (p = 0.099). At 24hpi, the percentage of externalized phosphatidyl-serines (early apoptosis) was significantly higher in haemocytes from animal injected with ASW (control) (26.63% ± 1.79) than that from OsHV-1 infected oysters (11.59% ± 1.31) (p<0.0001). At the same sampling time, the percentage of late apoptotic/secondary necrotic cells was also significantly higher in haemocytes from animal injected with ASW (3.45% ± 0.49) than that from OsHV-1 infected oysters (1.74% ± 0.11) (p = 0.001) and no significant difference between the two conditions was observed for primary necrotic/dead cells. At 48hpi, the percentage of early apoptotic cells was significantly higher in haemocytes from animal injected with ASW (24.41% ± 0.80) than that from OsHV-1 infected oysters (16.51% ± 2.29) (p = 0.001). On the contrary, the percentages of late apoptotic/secondary necrotic and primary necrotic/dead cells were significantly higher in haemocytes from OsHV-1 infected oysters (5.32% ± 0.47 and 1.59% ± 0.46, respectively) than those from animal injected with ASW (2.65% ± 0.10 and 0.55% ± 0.06, respectively) (p<0.0001 and, p = 0.01, respectively).

The percentage of externalized phosphatidyl-serine in haemocytes from oysters injected with ASW increased from 2 to 24hpi (p = 0.022) and no significant variation of the late apoptotic/secondary necrotic and primary necrotic/dead cell percentages was observed at 2, 24, and 48hpi.

The percentages of early apoptotic, late apoptotic/secondary necrotic and primary necrotic/dead cells in haemocytes from OsHV-1 infected oysters decreased from 2 to 24hpi (p = 0.002, p = 0.003, and p = 0.033, respectively) and then increased from 24 to 48hpi (p = 0.021, p<0.0001, and p = 0.007, respectively). However, the percentage of late apoptotic/secondary necrotic cells was significantly higher at 48hpi than that observed at 2hpi (p<0.0001).

**Fig 7:**
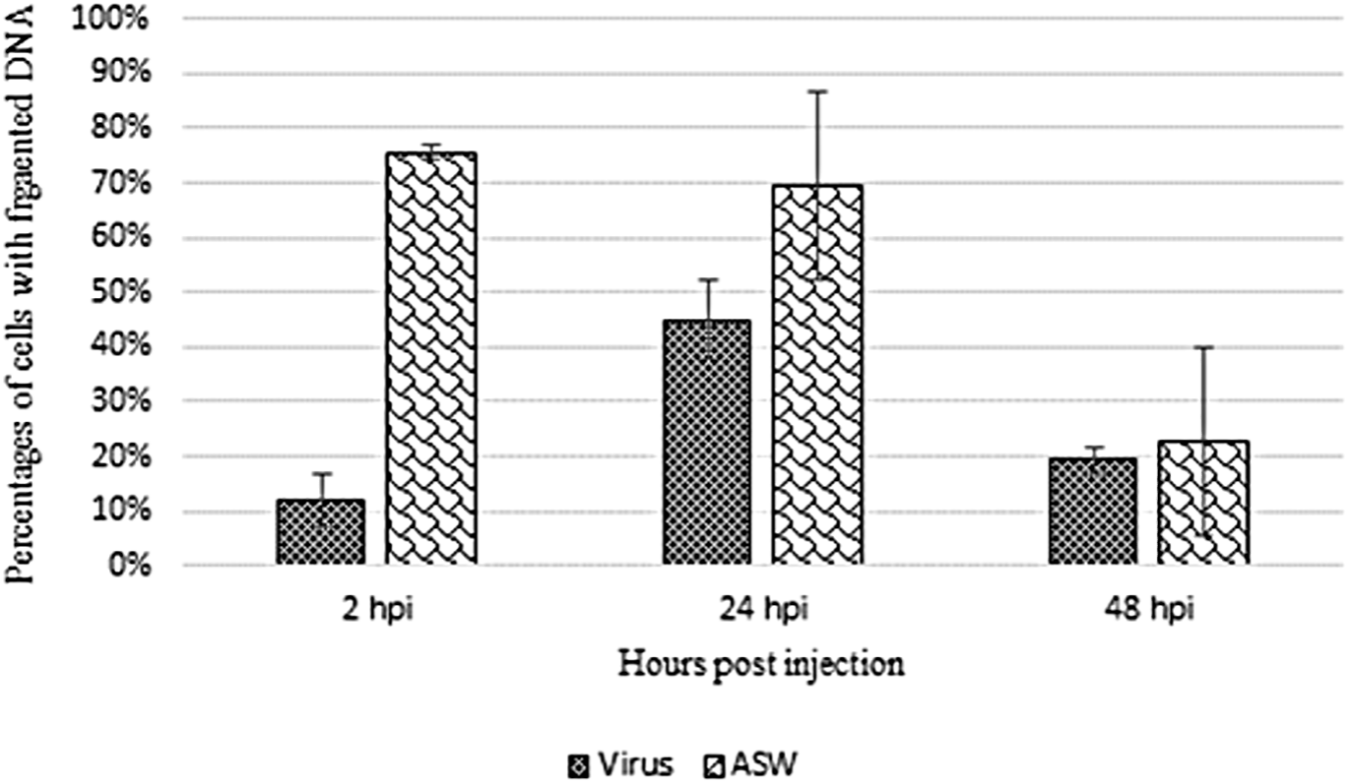
Detection of cells with fragmented DNA in haemocytes from OsHV-1 infected and control oysters by TUNEL assay. Percentages of cells with fragmented DNA in haemocytes from OsHV-1 infected (virus) and control oysters (ASW) at 2, 24, and 48hpi.

**Fig 8:**
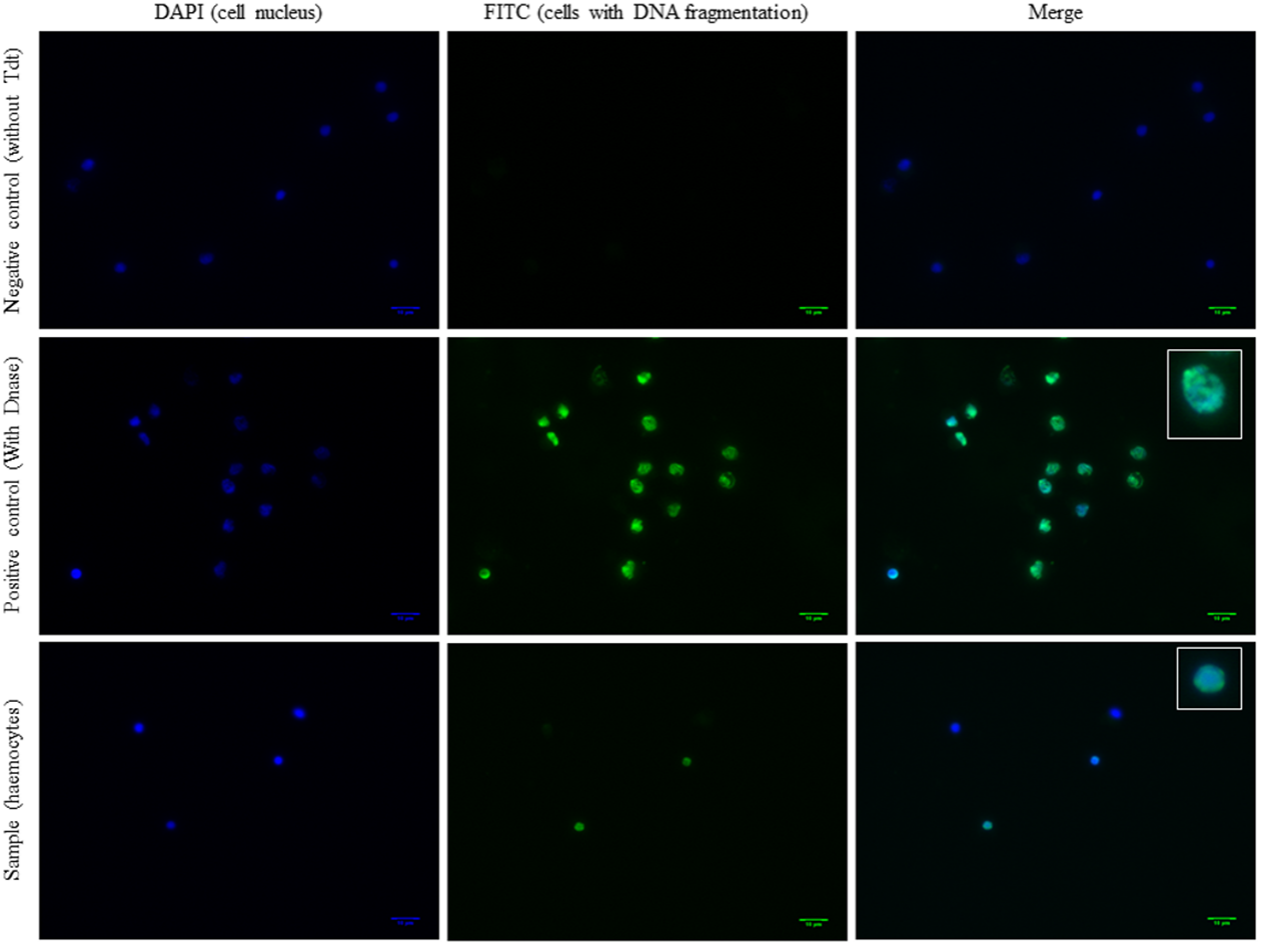
Detection of cells with fragmented DNA in haemocytes by epifluorescence microscope (FITC) from OsHV-1 infected and control oysters (TUNEL assay). Negative control consisted in slides without the Terminal deoxynucleotidyl transferase (Tdt) and positive control corresponded to cells treated with the DNase.

The TUNEL assay was carried out on haemocytes from OsHV-1 infected and ASW injected oysters sampled at 2, 24, and 48hpi (Fig 7 and Fig 8). The percentage of cells with DNA fragmentation would be higher in haemocytes from oysters injected with ASW than that in haemocytes from OsHV-1 infected oysters at 2hpi. At 24 and 48hpi, the percentages of labelled cells between the two conditions were not different.

### Apoptosis modulation at molecular level: Relative gene expression of host genes involved in the apoptosis pathway

**Fig 9:**
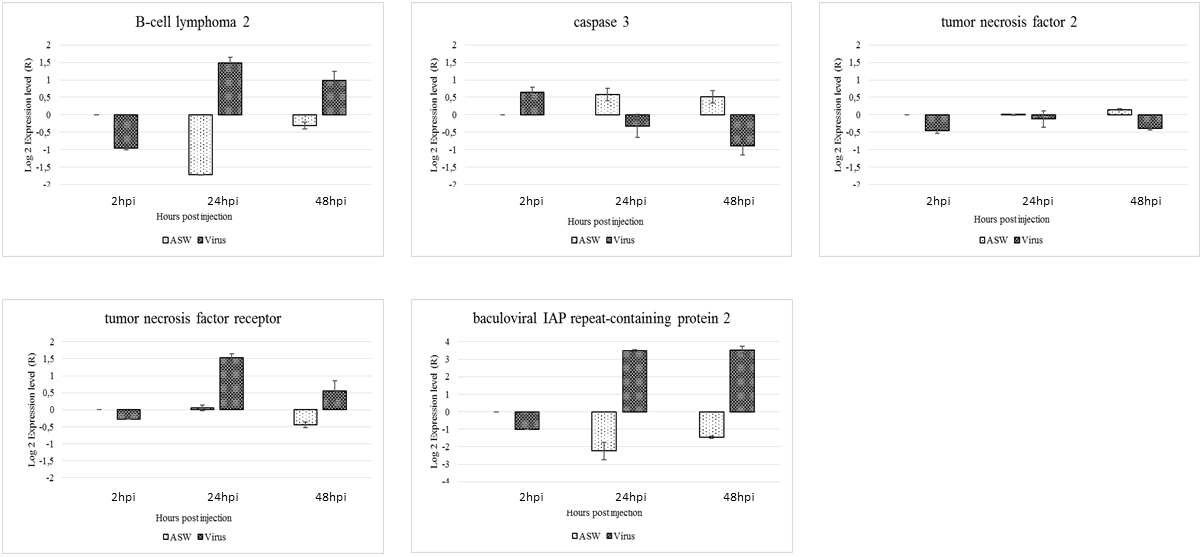
Relative expression of five apoptosic genes in haemoctes from OsHV-1 infected (virus) and control oysters (ASW): B*-*cell lymphoma 2, caspase 3, tumor necrosis factor 2, tumor necrosis factor receptor, baculoviral IAP repeat-containing protein 2.

The RNA level was reported for five *C*. *gigas* genes (Bcl-2, IAP-2, TNF-2, TNFR, and Casp-3) related to the apoptotic pathway in haemocytes from OsHV-1 infected and ASW injected oysters (Fig 9). The RNA level of Bcl-2 and IAP-2 genes in haemocytes from OsHV-1 infected oysters collected at 2hpi and in haemocytes from oysters injected with ASW sampled at 24 and 48hpi, was down-regulated. The RNA of Casp-3 gene was up-regulated at the same times. The RNA level of TNF-2 gene was down-regulated in haemocytes from OsHV-1 infected oysters collected at 2, 24, and 48hpi whereas this one was slightly up-regulated in haemocytes from oysters injected with ASW sampled at 24 and 48hpi. The RNA level of TNFR gene in haemocytes from OsHV-1 infected oysters was down-regulated at 2hpi and then up-regulated at 24 and 48hpi. In haemocytes from OsHV-1 infected oysters, the expression level of genes involved in the inhibition of the apoptotic pathway (Bcl-2 and IAP-2) and those participating to the apoptosis (Casp-3 and TNFR) were up-regulated and down-regulated, respectively, at 24 and 48hpi. On the contrary, the expression level of Bcl-2 and IAP-2 was down-regulated and that of Casp-3 was up-regulated in haemocytes from oysters injected with ASW at 24 and 48hpi.

## Discussion

OsHV-1 DNA, RNA, and protein detection was monitored through real time PCR, real time RT PCR, and immunofluorescence assay (IFA), respectively, in order to demonstrate viral infection in experimentally infected oysters, particularly in haemocytes. Cumulative mortality rates recorded in the two oyster batches (74% and 100%), were associated with high OsHV-1 DNA amounts in haemocytes and mantle from OsHV-1 infected oysters, suggesting high viral replication, as previously reported by Schikorski *et al*. [54,55]. Despite significantly different mortality rates between the two oyster batches, which can be linked to their age, no significant difference of the viral DNA quantity from mantle was observed between the two batches for each sampling time. However, the detection of low viral DNA amounts (below the quantifiable limit) and viral RNA in some oysters of the J6 batch injected with ASW, suggests that these oysters may have been previously exposed to OsHV-1. This is a recurrent issue and this detection may correspond to either a persistent or recently acquired infection [31,35,39,56,57]. In addition, the hypothesis that viral replication occurred in haemocytes was suggested by the detection of OsHV-1 transcripts and proteins in these cells from OsHV-1 infected animals at 24 and 48hpi. Among the five targeted OsHV-1 ORFs, the ORFs 25, 72, 75, and 87 were more expressed at 24 and 48hpi, as previously described by Morga *et al*. (pers. comm.) during *in vitro* assays by incubating haemocytes and the virus. These results showed that some OsHV-1 transcripts were detected inside Pacific oyster haemocytes suggesting than OsHV-1 is capable to penetrate in this cell type. In addition, the early detection of viral transcripts at 2hpi and the high increase of viral transcript amounts from 2 to 48hpi in haemocytes from OsHV-1 infected oysters, indicate that the entry of the virus into cells was rapid and quickly followed by effective viral RNA transcription. These observations are in accordance with previous results obtained by Segarra *et al*. [36] where viral transcripts were detected in the mantle as early as 4hpi and expression levels of 38 viral genes peaked at 26hpi during an experimental infection trial with OsHV-1. Rosani *et al*. [34] performed a dual RNA-seq analysis on OsHV-1 infected spat and notably noticed that most expression viral ORFs encoded putative membrane proteins.

IFA assay showed for the first time the presence of a putative viral apoptosis inhibitor protein in haemocytes from experimentally OsHV-1 infected oysters at 24 and 48hpi, suggesting that the virus can modulate the host apoptosis. This phenomenon was notably observed for pseudorabies virus (PRV) which is able to block apoptosis in infected trigeminal ganglionic neurons during acute infection of swine [58].

TEM examination of haemocytes from OsHV-1 infected oysters sampled before the first oyster death, allowed the observation of viral capsids in the nucleus of very few cells interpreted as haemocytes. Haemocytes were not previously reported as target cells for OsHV-1, contrary to fibroblast-like cells and cardiomyocytes [59,60]. No enveloped particles were reported through TEM examination suggesting that haemocytes would not be a privileged location for the viral particle production. The OsHV-1 DNA, RNA, and protein detection in haemocytes seems to indicate that the virus might initiate its replication cycle in these cells without reaching enveloped particle production. Different types of viral infection propagation are used by herpesviruses including herpes simplex virus, involving extracellular free particles and cell-to-cell spread where naked virions contained in one cell are transmitted directly to the next cells via cell junctions [61–64]. In addition, Silva *et al*. [65] demonstrated that a human cytomegalovirus mutant, unable to produce wrapped infectious particles (just capsids coated with tegument proteins), can spread from cell-to-cell. In this context, we hypothesize that OsHV-1 capsids may be transmitted from haemocytes to other cells present in different organs (mantle, gills, heart) which are likely viral replication sites. However, the question remains open concerning the role of this cell type in virus dissemination throughout the oyster body.

The presence of four ORFs encoding putative apoptosis inhibitor in the OsHV-1 genome had led to the hypothesis that OsHV-1 might modulate the *C*. *gigas* apoptosis. In this context, cellular and molecular investigation was conducted on haemocytes from OsHV-1 infected and ASW injected oysters. The early apoptosis marker, corresponding to cytoplasmic membrane modification, indicated that more phosphatidyl-serines were externalized in haemocytes collected from ASW injected oysters than in haemocytes from OsHV-1 infected oysters at 24 and 48hpi, suggesting that the apoptosis might be inhibited in presence of OsHV-1 in this cell type. OsHV-1 might first inhibit apoptosis to allow its own replication. At 48hpi, the percentages of late apoptotic/secondary necrotic cells and primary necrotic/dead cells were higher in haemocytes from OsHV-1 infected oysters than in haemocytes from oysters injected with ASW. This could be related to the host itself to regulate the apoptotic process, since *C*. *gigas* have a high number of genes related to apoptosis regulation (48 potential IAPs) [66]. Particularly, haemocytes could be a cell type inside which the viral replication is not fully realized. Results seemed to indicate a confrontation between the host, which initiated the apoptotic process to reduce viral spread, and the virus, which inhibited apoptosis to facilitate its own replication cycle, as notably observed for HSV-1 infection [50].

The final apoptosis stage consists in cellular DNA fragmentation which has been investigated by TUNEL assay. These first results seem to show more cells with DNA fragmentation in haemocytes from oysters injected with ASW in comparison with haemocytes from OsHV-1 infected oysters at 24 and 48hpi and led hypothesize an inhibition of the apoptotic process in presence of OsHV-1. Repeated experiments appear necessary to confirm these observations.

One of the objectives of the present study was to investigate apoptosis modulation at molecular level by targeting five apoptosis related genes in haemocytes from oysters after an OsHV-1 challenge and haemocytes from oysters injected with ASW. The relative gene expression showed that genes encoding the TNF (ligand activating the extrinsic apoptotic pathway) and the Caspase 3 (protein playing a dominant role in the apoptosis execution phase) were under-expressed whereas genes encoding the TNFR and Bcl-2 (anti-apoptotic protein involved in the inhibition of release of cytrochrome c from mitochondria) were over-expressed in haemocytes from OsHV-1 infected oysters in comparison with haemocytes from ASW injected ones. The up-regulation of the gene encoding Bcl-2 was previously reported by [33] in haemocytes in presence of OsHV-1 during *in vitro* assays. In addition, [32] demonstrated that several genes involved in the inhibition of apoptosis, were up-regulated in experimentally infected spat, such as Birc7. The up-regulation of the TNFR gene expression was surprising as TNFR is involved in signal transduction after TNF recognition resulting in apoptosis as previously mentioned. However, it has been also reported that TNFR can be present not only on the cell membrane but also free and may inhibit apoptosis by interacting with TNF [67]. This apoptosis modulation might be initiated by the host itself or by the virus by producing apoptosis-inhibiting factors which might interfere with the involvement of Bcl-2 gene family members and apoptotic pathway, as proposed by Du *et al*. [68] for the bovine herpesvirus type 1 (BHV-1). The oyster IAP gene, previously targeted by Segarra *et al*. [36] was over-expressed at 24 and 48hpi in haemocytes from OsHV-1 infected oysters in comparison with haemocytes collected from ASW injected oysters. These results are in agreement with those obtained by Segarra *et al*. [36] which reported that IAP cellular transcripts were up-regulated in families A (highly susceptible to OsHV-1 infection) and P (less susceptible to OsHV-1 infection) from 12hpi to 26hpi and from 26hpi to 72hpi, respectively.

**Fig 10:**
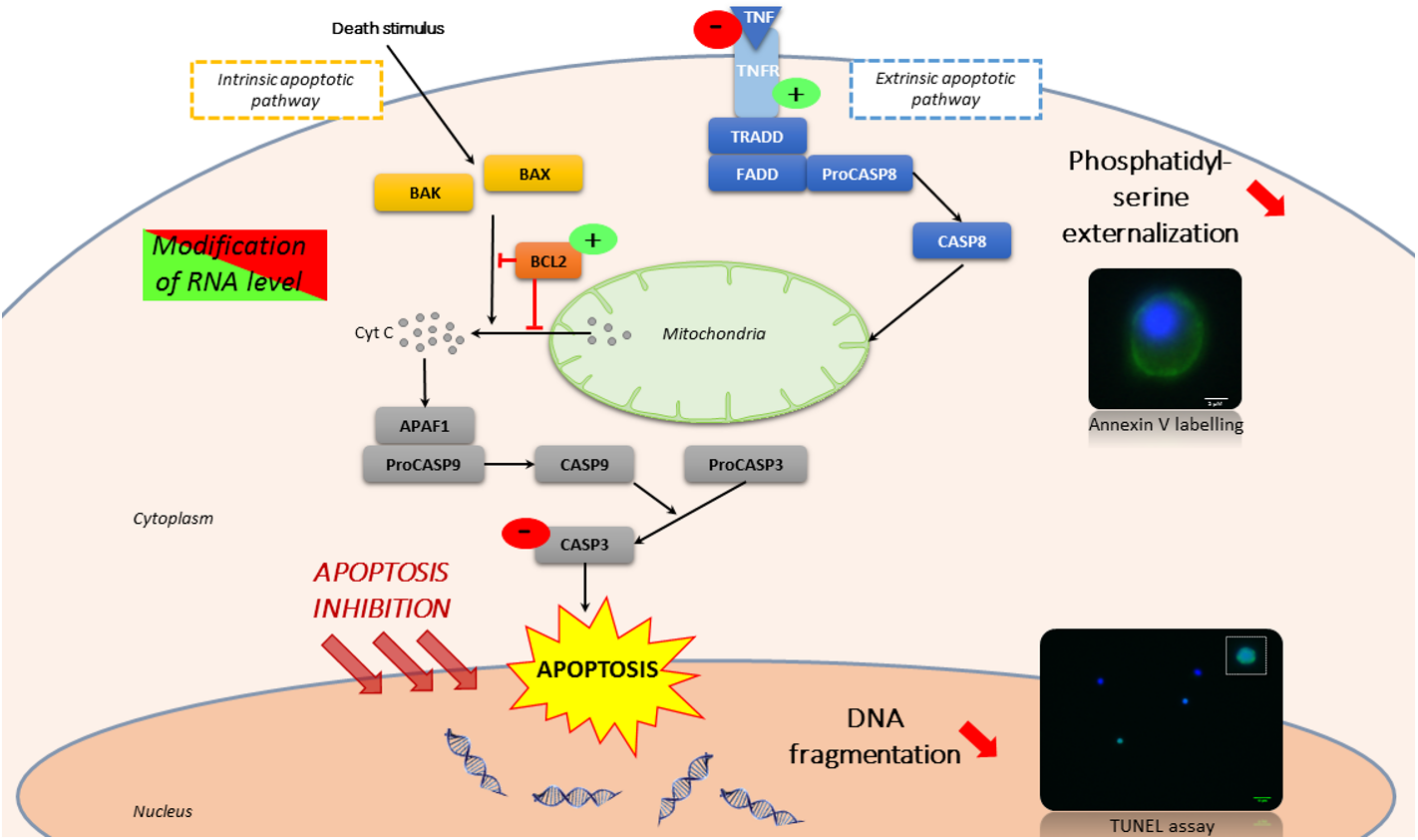
OsHV-1 detection in haemocytes from experimentally infected oysters in relation with inhibition of apoptosis.

Taken together, these results showed that anti-apoptosis phenomenon appears to be important in haemocytes from OsHV-1 infected oysters which may be directly promoted by the virus or induced by the host to modulate the apoptosis (Fig 10). In this way, Jenner *et al*. [69] suggested that may exist a balance between pro-apoptosic and anti-apoptosic factors and consequently, the apoptosis modulation appears to be a complex pathway, controlled at several regulation levels.

Recent study showed that several human herpesvirus including HSV-1 may initiate replication in response to host cell apoptosis, suggesting that an alternative apoptosis-triggered replication program might be a general feature of herpervirus biology [70]. In this way, Du *et al*. [68] reported that two pro-apoptotic drugs (dexamethasone and 2[[3-(2,3-dichlorophenoxy) propyl]amino]-ethanol) accelerated viral gene expression in ganglionic organ cultures and accumulation of infectious viral particles. Apoptosis might be used by herpesviruses to exit from latency. Despite latency was not still clearly demonstrated for OsHV-1, it would be interesting to explore this way by using pro-apoptotic drugs on experimentally infected and uninfected oysters with different molecular and cellular approaches.

As the viral infection rapidly occurs, shorter sampling interval between 2 and 24hpi would be considered to better describe the apoptosis modulation in oyster tissues and haemocytes. Additionally, Morga *et al*. (pers. comm.) recently developed an *in vitro* assay by incubating *C*. *gigas* haemocytes with viral suspension until 72h. This approach would be of interest to study the host genes involved in programmed cell death (i.e. apoptosis and autophagy). Similar tool was also previously used by Gervais *et al*. (pers. comm.) to examine the response of *O*. *edulis* confronted to the protozoan parasite, *Bonamia ostreae*. The diversity of the host apoptotic response might be investigated using different oyster pathogens (bacteria or parasites) and OsHV-1 variants, in combining the analysis of several apoptosis markers or host gene expression, such as the nuclear factor kappa B kinase beta (IkB2), since some viruses can activate NF-kB to block apoptosis [71]. In the present work, apoptosis was investigated at the transcriptional and the cellular level and might be complete by approaches allowing the study of post translational apoptosis, as previoulsy used for urchin [72].

## Materials and methods

### Experimental design

Two *Crassostrea gigas* batches with different ages were used for the experimental infections by OsHV-1: NSI 01/15 (14 months old) and J6 (12 months old). These batches, NSI 01/15 and J6, were produced in 2014 at the Ifremer’s hatcheries located in Argenton (Brittany, France) and La Tremblade (Poitou Charente, France), respectively, before to be reared at the Ifremer’s facilities in Bouin (Vendée, France). Viral challenges were performed according to the protocol developed by Schikorski *et al*. [54,55]. Briefly, 100μL of tissue homogenate from OsHV-1 experimentally infected oysters at 10^5^ viral DNA copies/μL were injected into the adductor muscle of each spat. For control oysters, 100μL of sterilized artificial seawater (ASW) were injected into the adductor muscle of spat. Seventy two oysters (J6) were distributed in 8 tanks (9 animals per tank) and 184 oysters (NSI 01/15) were placed in 8 other tanks (23 animals per tank). Among the 8 tanks of each oyster batch, 4 of them contained spat injected with tissue homogenate and 4 other tanks were dedicated for spat injected with ASW. At 2, 24, and 48 hours post injection (hpi), all animals of one tank for each condition (OsHV-1 infected oysters and ASW injected ones) were used for analysis. The two remaining tanks of each oyster batch were dedicated for mortality monitoring during one week. For each condition and time sampling, the mantle of 5 animals were dissected for OsHV-1 DNA quantification and the hemolymph was withdrawn from the adductor muscle of all individuals with a 1mL syringe and a needle (0.40mm x 90mm), before to be pooled and filtered through a nylon grid of 70μM (352350, Dutcher) to eliminate large debries. Haemolymph was held on ice all the time to limit cell aggregation. Hemolymph of NSI 01/15 batch was used for OsHV-1 protein detection by IFA with polyclonal antibodies targeting a putative inhibitor protein and DNA fragmentation detection by TUNEL assay. Hemolymph of J6 batch was treated for OsHV-1 transcripts detection, relative expression of genes involved in the apoptosis pathway or its modulation, phosphatidyl-serine externalization by flow cytometry, and TEM examination (Table 1).

**Table 1:** Analysis performed on the two oyster batches (NSI 01/15 and J6).

Analysis	NSI 01/15	J6
Mortality monitoring	**×**	**×**
Viral DNA quantification in haemocytes and in mantle (real time PCR)	**×**	**×**
Viral transcript detection (real time RT PCR)	_	**×**
Viral protein detection (IFA)	**×**	_
Viral capsid detection (TEM)	_	**×**
Phosphatidyl-serine externalisation (flow cytometry)	_	**×**
DNA fragmentation (TUNEL)	**×**	_
Relative gene expression of host genes involved in the apoptosis pathway (real time RT PCR)	_	**×**

### Molecular analysis

#### OsHV-1 DNA quantification

The DNA was extracted from the mantle of 5 spat and 200μL of hemolymph sampled at 2, 24, and 48hpi for each condition (OsHV-1 infected spat and ASW injected ones) with the QIAmp DNA mini Kit (Qiagen), according to the manufacturer’s instructions. DNA extracted from the mantle was measured using a NanoDrop spectrophotometer 2000 (Thermo Fisher Scientifc) before to be diluted at 5ng/μL for optimal PCR reaction conditions. For haemolymph samples, the elution was performed in 50μL of water for molecular biology and 5μL of them were directly used for real time PCR analysis.

OsHV-1 DNA quantification was performed with the real time PCR based on TaqMan^®^ chemistry, targeting a putative apoptosis inhibitor [73]. Briefly, 5μL of DNA were added to the reaction mixture composed of 10μL of Brillant III Ultra-Fast QPCR Master Mix (Agilent Technologies), 0.4μL of each primers (20μM) OsHV1BF (forward) 5’-GTCGCATCTTTGGATTTAACAA-3’ and B4 (reverse) 5’-ACTGGGATCCGACTGACAAC-3’, 0.4μL of TaqMan^®^ probe (10μM) 5’-TexasRed-TGCCCCTGTCATCTTGAGGTATAGACAATC-BHQ2-3’, and 3.8μL of distilled water. The amplification was performed in duplicate for each sample and accomplished using a Mx3000P real time PCR thermocycler (Agilent). The PCR conditions were 1 cycle at 95°C for 3min, 40 cycles of amplification at 95°C for 10s, 60°C for 20s. Assays included a standard curve and a negative control (5μL of distilled water instead of the 5μL of sample DNA). Results were expressed in viral DNA copies in one nanogram of total DNA for oyster tissue samples (mantle) or in viral DNA copies per microliter of hemolymph.

#### OsHV-1 RNA detection and relative expression of oyster genes

The RNA extraction was performed on 5mL of haemolymph pool from experimentally infected and ASW injected spat sampling at 2, 24, and 48hpi. Total RNA was extracted using the Ambion^®^ TRIZOL^®^ Reagent™ (Life Technologies, Saint Aubin, France) according to the manufacturer’s recommendations. The RNA quality and quantity were controlled with NanoDrop spectrophotometer (Thermo Fisher Scientifc). A DNase treatment was performed with the Ambion^®^ TURBO DNA-free™ (Life Technologies, Saint Aubin, France) according to the manufacturer’s instructions. A second RNA extraction was then realized using TRIZOL^®^ Reagent™ to inhibit and eliminate the DNase.

To control the absence of oyster and virus genomic DNA after the DNAse treatment, a real time PCR (SYBR^®^ Green chemistry) targeting the oyster elongation factor alpha (EF1 alpha) was performed [33,36]. Five microliters of DNA were added to the reaction mixture composed of 10μL of Brillant III ultra-Fast 99 SYBR^®^ Green Master Mix 10X (Agilent Technologies), 2μL of each primer concentrated at 1.5μM, and 1μL of distilled water. The amplification was performed in duplicate for each sample and accomplished using a Mx3000P real-time PCR thermocycler (Agilent). The PCR conditions consisted of 1 cycle at 95°C for 3min, 40 cycles of amplification at 95°C for 5s, 60°C for 20s, and followed by a dissociation stage (95°C for 1min, 60°C for 30s, and 95°c for 30s).

After RNA quantification, first-strand cDNA synthesis was carried out using the Super-Script® III First-Strand Synthesis System (Invitrogen) from 500ng of RNA treated. A 1:30 dilution of the cDNA was realized and 5μL of them were used for PCR investigation.

Five viral genes encoding three putative membrane proteins (ORFs 25, 41, and 72), a putative dUTPase (ORF 75), and a putative apoptosis inhibitor (ORF 87) were targeted to evaluate the viral transcript production. These genes were previously used by Morga *et al*. (pers. comm.), Segarra *et al*. [36], and Martenot *et al*. (pers. comm.). The SYBR^®^ Green PCR conditions were the same as explained above, except the viral primer concentration which was used at 3μM. The OsHV-1 transcript number was represented by the following formula adapted from De Decker *et al*. [74]: 1/(Ct_viral ORF_−Ct _elongation factor_) (Morga *et al*., pers. comm.).

Five oyster cellular genes involved in the apoptosis pathway were selected: Bcl-2 [33], baculoviral IAP repeat-containing protein 2 (iap-2) [36], tumor necrosis factor 2 (TNF-2) [39], tumor necrosis factor receptor (TNFR) [39], and Caspase 3 (casp-3) [39]. The relative quantification value (ratio R) was calculated using the method described by Pfaffl [75]: R = [(E_target_)^ΔCT target(control-sample)^]/[(E_EFα-1_)^ΔCT EFα-1 (control-sample)^] and represented in Log base 2. Host gene expression was normalized to the elongation factor I (EF), as previously used by Renault *et al*. [33] and Segarra *et al*. [36]. The calibrator corresponded to haemocytes from oyster injected with ASW sampling at 2hpi.

### Double immunofluorescence assay (IFA)

#### Polyclonal antibody production and preparation for IFA

Polyclonal antibody targeting a putative apoptosis inhibitor encoded by ORF 87 was selected among three antibodies already used in immunohistochemistry (IHC) to detect OsHV-1 proteins, since satisfactory staining was obtained [57]. Antibody was produced by ProteoGenix (Schiltigheim, France) at 1mg/mL. Briefly, the partial cDNA of ORF 87 was cloned in pET-43.1a vector in order to express the protein with His tag in N-terminal position (cloning strategy: Ndel/XhoI). After purification, the recombinant protein was injected to two different rabbits. The post-immune sera of the rabbits were collected and polyclonal antibodies were separately purified using protein A affinity chromatography. This antibody was previously used by Martenot *et al*. [57] to describe the localization and the tissue distribution of OsHV-1 proteins during an experimental infection by the virus.

Antibodies were treated using proteins extracted from non-challenged oysters (considered as non-infected animals) to reduce non-specific epitope interactions [57]. Briefly, antibodies (15μL) were incubated with 110μL of proteins extracted from non-challenged oysters (20mg/mL) and 300μL of Tris-Buffered Saline with additional 0.1% Tween 20 (TBST) under gentle stirring at 4°C overnight. After incubation, the mixture was centrifuged at 16,000g during 15min at 4°C and the supernatant was placed in a Falcon^®^ tube. To obtain final primary antibody concentration of 1:100, 1,075μL of 1X PBS with 1% of bovine serum albumin (BSA) (A7906, Sigma-Aldrich) were added to the supernatant containing anti-viral antibodies. This solution was then left on haemocytes during the immunofluorescence assay.

#### Immunofluorescence assay protocol

Three hundred microliters (100μL per well) of haemolymph were deposited on glass slide and centrifuged at 100×g during 1min at 4°C. The supernatant was removed and cells were fixed with ice cold methanol for 30s. After two washes in 1X PBS (5min and 15min, respectively), potential sites of non-specific interaction were blocked using 5% BSA dissolved in 1X PBS during 1h at room temperature. Samples were washed three times in 1X PBS for 10min each. The slides were incubated overnight at 4°C with the two primary antibodies targeting the putative OsHV-1 IAP (1:100) and the actin (1:500) (A4700, Sigma) diluted in 1X PBS supplemented with 1% of BSA. Unbound primary antibodies were removed by three washes in 1X PBS. Primary antibodies were detected using two fluorochrome-conjugated secondary antibodies (1:400) diluted in 1X PBS with 5% of BSA for 45min at room temperature in the dark. The secondary antibodies conjugated with the dylight anti-rabbit 488 (072-03-15-06, Eurobio) and the dylight anti-mouse 549 (DI-2549, Eurobio), were used for viral proteins and oyster actin detection, respectively. After two washes in 1X PBS (5min), samples were mounted using ProLong^®^ Gold antifade mountant with 4’,6-diamidino-2-phenylindole (DAPI) (P36935, ThermoFisher Scientific). Samples were examined under fluorescence microscope (Leica DFC3000 G).

### Transmission electron microscopy (TEM) examination

Three milliliters of haemolymph from OsHV-1 experimentally infected oysters and ASW injected spat sampling at 2, 24, and 48hpi were centrifuged at 1,500×g during 10min at 4°C to obtain haemocyte pellets and the supernatant was eliminated. Samples were fixed in 3% glutaraldehyde solution (200μL) for one day at 4°C. Cells were washed in 0.4M cacodylate buffer. After dehydratation in successive baths of ethanol, two baths of propylene oxide were performed and then samples were progressively impregnated and embedded in Epon [60]. After polymerization at 60°C, semi-thin sections were cut at 1μM thickness for quality control and then to 80–85nm before to be floated onto copper EM grids and stained with uranyl acetate and lead citrate [60]. Samples were examined using a transmission electron microscope (JEOL-JEM 1000) at 60 kVolts.

### Phosphatidyl-serine externalization measured by flow cytometry

Three replicates of haemolymph (200μL) for each condition and sampling time were used to measure the phospholipid asymetry of plama membrane with the Annexin V-FITC apoptosis detection kit (ABC500FI, Eurobio), as previously used by Gervais *et al*. [51]. After centrifugation at 1,300×g during 10min at 4°C, the supernatant was eliminated and 190μL of 3X Binding buffer were added to suspend the haemocyte pellet. Ten microliters of Annexin-V-FITC were added to cell suspensions and incubated for 20min at room temperature in the dark. After centrifugation at 1,300×g during 10min at 4°C, the supernatant was removed and cells were suspended in 185μL of 3X Binding buffer. Fifteen microliters of propidium iodide (PI) concentrated at 20μg/mL were added. Samples were kept on ice in the dark until flow cytometry analysis (EPICS XL 4, Beckman Coulter) with settings previously determined by Morga *et al*. [76] and Gagnaire *et al*. [77–79]. Cells should be separated in four groups: (1) FITC Annexin-V positive and PI negative (early apoptosis), (2) FITC Annexin-V and PI positive (end stage apoptosis or secondary necrosis), (3) FITC Annexin-V and PI negative (alive cells), and (4) FITC Annexin-V negative and PI positive (primary necrosis/dead cells). The percentages of apoptotic cells were defined as the ratio: number of cells labelled with Annexin-V / number of observed cells.

### DNA fragmentation (TUNEL)

Three hundred microliters (100μL per well) of haemolymph were deposited on glass slide (duplicate) and centrifuged at 100×g during 1min at 4°C. The supernatant was removed and cells were fixed with 4% paraformaldehyde (818715, Merck) for 10min at room temperature. Cells were hydrated in 1X PBS for 5min and were then permeabilized by heating slides in 0.1M citrate buffer at pH6 in a microwave (350W) for 5min [80]. The DNA fragmentation was detected using the *In situ* Cell Death Detection kit (Roche) according to the manufacturer’s recommendations except that enzyme solution was half diluted [51]. Negative controls consisted in incubated slides without the Terminal deoxynucleotidyl transferase (Tdt) and positive controls corresponded to cells treated with the Ambion^®^ TURBO DNA-free™ DNase (AM2239, Life Technologies, Saint-Aubin, France) during 10min at 37°C. Samples were mounted using ProLong^®^ Gold Antifade Mountant with DAPI (P36935, ThermoFisher Scientific). Samples were examined under fluorescence microscope (Leica DFC3000 G).

### Statistical analysis

Statistical analyses were carried out using the XLSTAT-Pro^®^ 2014.5.03 software (Addinsoft, Paris, France) and the normality of data was tested using a Shapiro-Wilk test.

The comparison of OsHV-1 DNA amounts in the mantle between the two batches was performed using a parametric t-test or a non-parametric Mann-Whitney test if normal distribution could not be demonstrated.

Flow cytrometry data were compared between the two conditions (haemocytes from OsHV- 1 infected and ASW injected oysters) by a two-way analysis of variance (ANOVA) followed by a Tukey post-test.

## Conclusion

For the first time, the apoptosis response of *C*. *gigas* was investigated by combining various approaches at molecular and cellular levels in haemocytes collected from experimentally infected and control oysters in laboratory conditions. Taken together, the down-regulation of genes encoding proteins involved in the activation of the apoptotic pathway and the up-regulation of genes encoding anti-apoptotic proteins showed an important anti-apoptosis phenomenon in haemocytes from OsHV-1 infected oysters. In addition, results obtained from cellular investigation based on the detection of phosphatidyl-serine externalization by flow cytometry reinforced these findings. However, the *C*. *gigas* response to an OsHV-1 infection appears to be complex and observed effects in haemocytes may be directly promoted by the virus or induced by the host himself to modulate the apoptosis.
